# Supplementary material for: Double-stranded RNA virus outer shell assembly by bona fide domain-swapping
Source: Nat Commun. 2017 Mar 13;8:14814. doi: 10.1038/ncomms14814 (PMC5355851; doi:10.1038/ncomms14814)
Supplement: Supplementary Information — Supplementary Figures [file ncomms14814-s1.pdf]

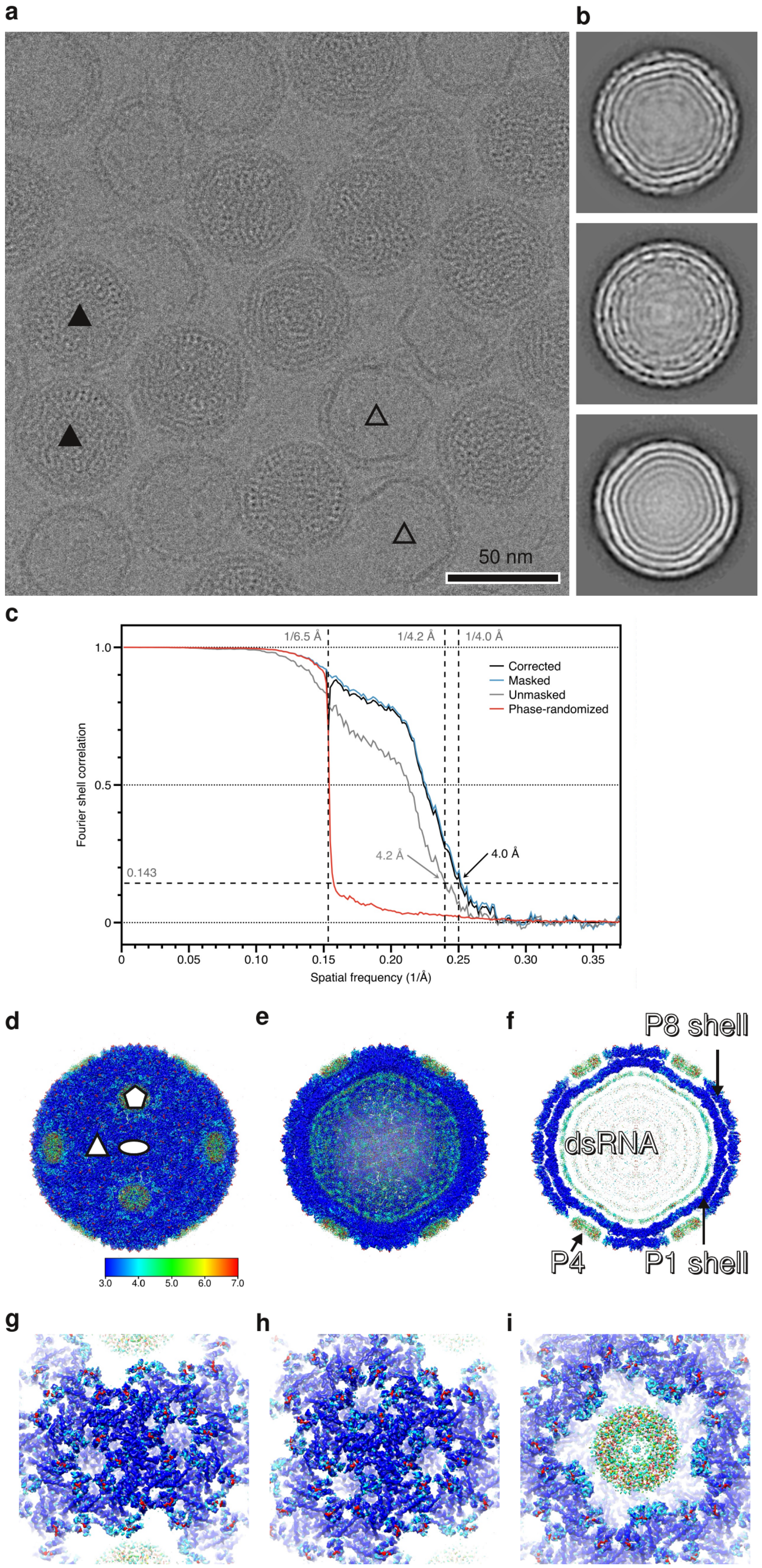

a
b
50 nm
c
1/6.5 Å
1/4.2 Å
1/4.0 Å
1.0
0.5
0
0.143
Fourier shell correlation
Corrected
Masked
Unmasked
Phase-randomized
4.2 Å
4.0 Å
0
0.05
0.10
0.15
0.20
0.25
0.30
0.35
Spatial frequency (1/Å)
d
e
f
P8 shell
dsRNA
P4
P1 shell
3.0
4.0
5.0
6.0
7.0
g
h
i

**Supplementary Figure 1 | Cryo-EM and resolution of the ϕ6 nucleocapsid.** (**a**) Micrograph of purified ϕ6 nucleocapsids (NCs). In addition to intact particles (filled triangle), empty broken particles can be seen (open triangles). Scale bar, 50 nm. (**b**) Three class averages are shown, revealing a multi-layered structure of the particle. In some classes (bottom), the outermost layer was partially missing. (**c**) Fourier shell correlation (FSC) calculated between two half-maps as a function of spatial frequency is plotted. FSC is plotted for the original, unmasked half-maps (grey) and masked half-maps having most of the RNA density removed (blue). FSC is also shown for phase-randomised half-maps (red) where phases were randomised at frequencies higher than 1/6.5 Å. The correlation drops at the cut-off frequency sharply below the noise threshold (0.143) as expected. The phase-randomization test was used to take into account the effect masking on the half-maps before calculating the final FSC curve (black). Good agreement between the masked and corrected curves indicated no adverse effects from masking. The corrected curve drops below the noise threshold at 1/4.0 Å indicating resolution of 4.0 Å in the reconstruction. (**d**) A surface rendering of the ϕ6 nucleocapsid reconstruction is shown. The surface is coloured based on the local resolution as indicated in the colour key (values in Å). One five-fold (pentagon), three-fold (triangle) and two-fold (ellipse) axes of symmetry are indicated. (**e**) Same rendering as in *d* but the front half of the volume is removed. (**f**) Same rendering as in **d**, but only a central slab of density is shown. Different structural components are labelled. (**g–i**) A close-up is shown along the two-fold **g**, three-fold **h**, and five-fold **i** axis of symmetry. Some surface exposed loops in the P8 shell, in addition to the components that do not follow icosahedral symmetry (P4 and dsRNA), are at a lower resolution than the well-ordered P1 and P8 shells.

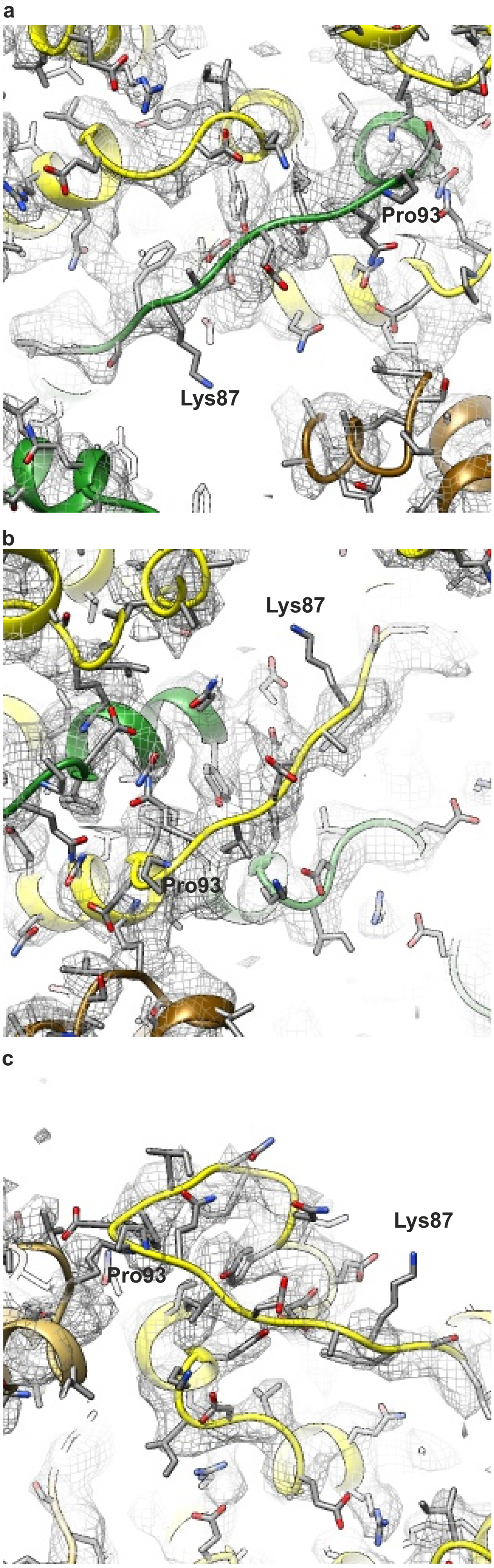
a
Pro93
Lys87
b
Lys87
Pro93
c
Lys87
Pro93

**Supplementary Figure 2 | Two conformations of the P8 linker region.** (**a–c**) The linker region is shown for two chains of P8 in the open conformation (**a** and **b**) and for one chain in the closed conformation (**c**). Amino acid side chain carbon atoms are coloured in grey, oxygen atoms in red and nitrogen atoms in blue. The protein backbone is show as a ribbon representation, coloured as in the main text Figure 2. Lys87 and Pro93 residues of P8 are labelled. Density for Lys87 was absent, probably due to radiation damage.

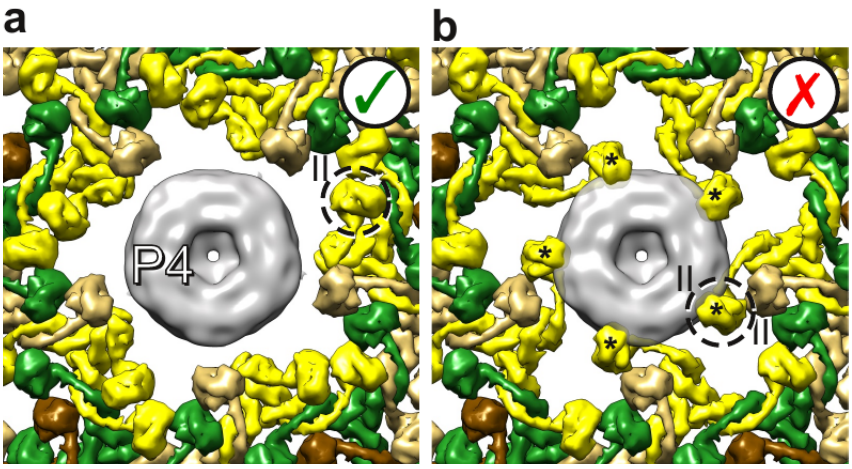


**Supplementary Figure 3 | Steric clashes of the P8 trimer Q in a hypothetical open conformation.** (**a**) A closed, compact conformation was observed in the type Q P8 trimers in the ϕ6 nucleocapsid reconstruction. In this conformation, no clashes occur between the P8 chains (yellow, gold, green, and brown) and the P4 hexamer (grey, labelled), as the $P8_Q$ domains (yellow, one circled) that are the closest to the hexamer fold away from it, clustering around the type II holes *via* homotypic interactions (a yellow P8 peripheral domain interacting with another 'yellow' P8 peripheral domain). (**b**) In a hypothetical case, significant clashes (asterisks) would occur between the peripheral domains (yellow, one circled) of the $P8_Q$ trimers (yellow) and the P4 hexamer density if the corresponding P8 chains were in an open conformation. One type II hole is labelled in **a** and **b**.

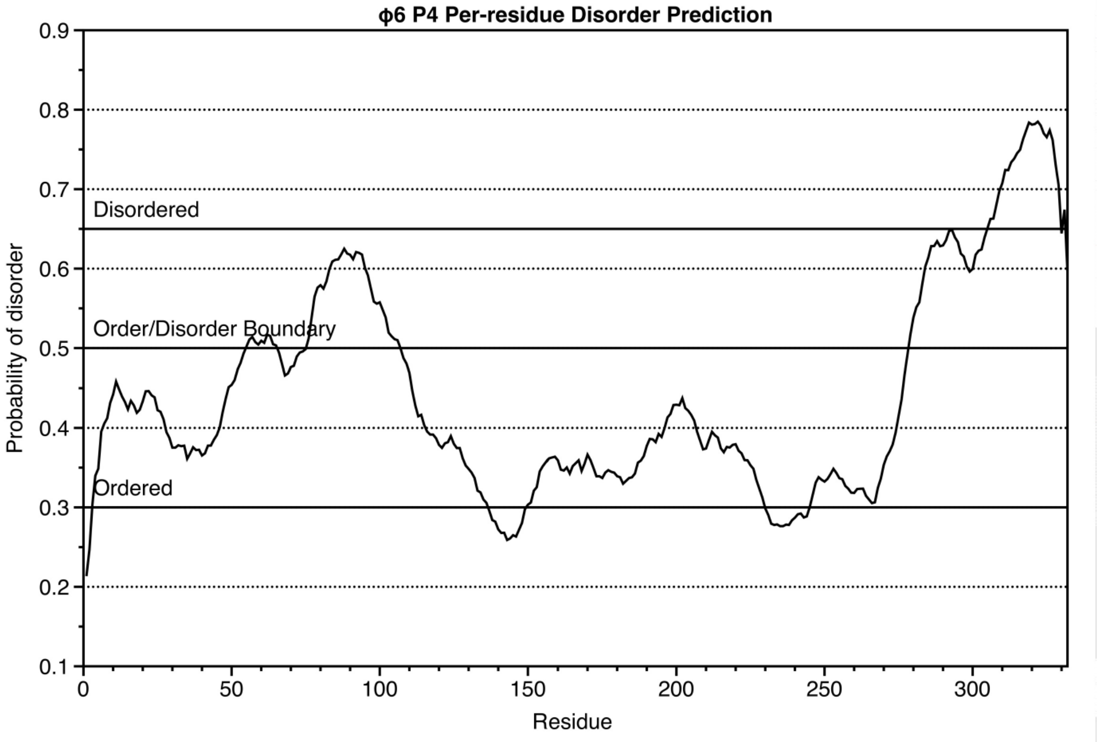


**Supplementary Figure 4 | Disorder prediction of ϕ6 P4.** The predicted probability of disorder is plotted as a function of residue number for ϕ6 P4. Analysis was performed in RONN and thresholds for predicted ordered/disordered regions are indicated.

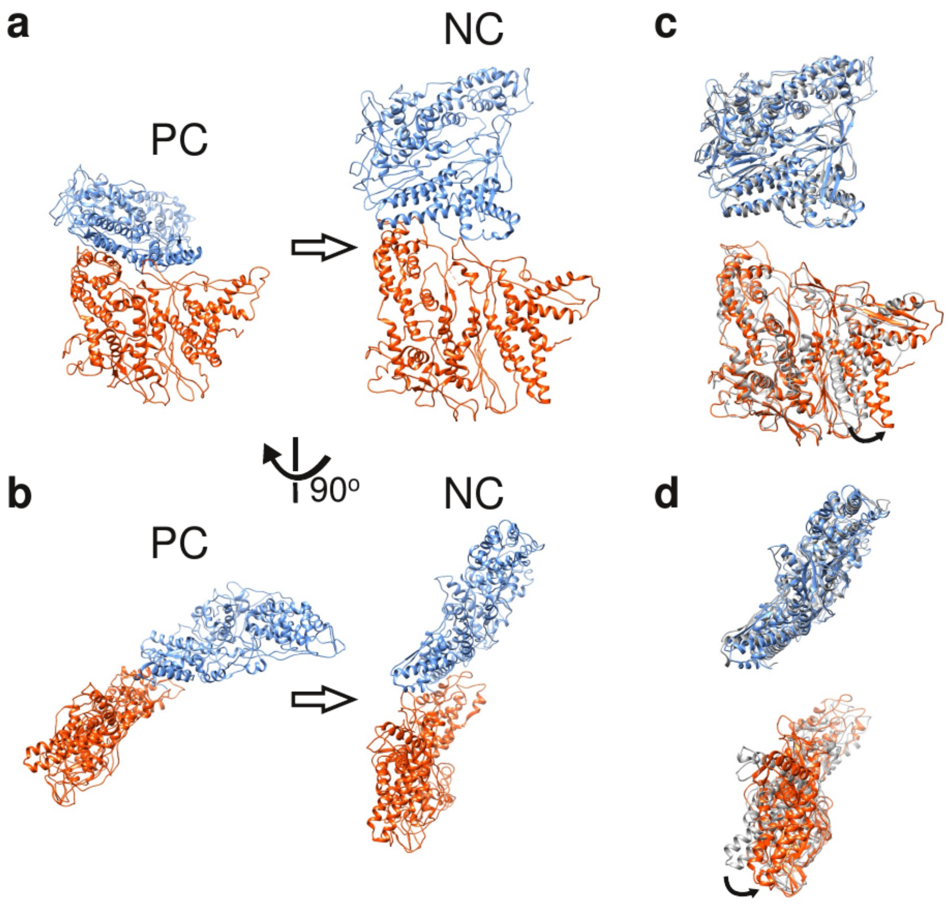


**Supplementary Figure 5 | Conformational changes in the P1 dimers upon expansion of the polymerase complex into a nucleocapsid core.** (**a–b**) The $P1_A$ (blue) and $P1_B$ (red) subunits are shown in the conformations observed in the polymerase complex (PC) and nucleocapsid (NC). The direction of the conformational change is indicated with an arrow. The view in **b** is the same as in **a** but rotated 90 degrees as indicated. (**c–d**) A structural alignment of $P1_A$ and $P1_B$ chains in their PC (grey) and NC conformations (blue and red). Root-mean-square deviation calculated between C-alpha atoms for the two different conformations of $P1_A$ and $P1_B$ was 2.6 Å and 6.0 Å, respectively. The movement of the $P1_B$ 'lever domain', the main conformational change occurring during expansion, is indicated with an arrow.
